# Endophytes, a Potential Source of Bioactive Compounds to Curtail the Formation–Accumulation of Advanced Glycation End Products: A Review

**DOI:** 10.3390/molecules27144469

**Published:** 2022-07-13

**Authors:** Lory Sthephany Rochín-Hernández, Lory Jhenifer Rochín-Hernández, Luis Bernardo Flores-Cotera

**Affiliations:** 1 Department of Biotechnology and Bioengineering, Cinvestav-IPN, Av. Instituto Politécnico Nacional 2508, Col. San Pedro Zacatenco, México City 07360, Mexico; lory.rochin@cinvestav.mx; 2 Department of Biomedicine and Molecular Biology, Cinvestav-IPN, Av. Instituto Politécnico Nacional 2508, Col. San Pedro Zacatenco, México City 07360, Mexico; lory.rochinh@cinvestav.mx

**Keywords:** diabetes, protein glycation, AGEs detoxification, carboxymethyl-lysine, RAGEs antagonists, methylglyoxal, endophytic metabolites

## Abstract

Endophytes, microorganisms that live in the internal tissues and organs of the plants, are known to produce numerous bioactive compounds, including, at times, some phytochemicals of their host plant. For such reason, endophytes have been quoted as a potential source for discovering bioactive compounds, particularly, of medical interest. Currently, many non-communicable diseases are threatening global human health, noticeably: diabetes, neurodegenerative diseases, cancer, and other ailment related to chronic inflammation and ageing. Intriguingly, the pathogenesis and development of these diseases have been linked to an excessive formation and accumulation of advanced glycation end products (AGEs). AGEs are a heterogeneous group of compounds that can alter the conformation, function, and lifetime of proteins. Therefore, compounds that prevent the formation and consequent accumulation of AGEs (AntiAGEs compounds) could be useful to delay the progress of some chronic diseases, and/or harmful effects of undue AGEs accumulation. Despite the remarkable ability of endophytes to produce bioactive compounds, most of the natural antiAGEs compounds reported in the literature are derived from plants. Accordingly, this work covers 26 plant antiAGEs compounds and some derivatives that have been reported as endophytic metabolites, and discusses the importance, possible advantages, and challenges of using endophytes as a potential source of antiAGEs compounds.

## 1. Introduction

Endophytes are microorganisms, mainly fungi and bacteria, that live at least during part of their life cycle within the internal tissues and organs of plants [1]. Endophytes are known to produce several bioactive compounds of pharmaceutical, agricultural, and industrial importance [2,3].

According to the World Health Organization, approximately 56.8% of the annual global deaths come from the most prevalent non-communicable diseases (NCDs): cardiovascular and respiratory diseases, cancer, and diabetes [4]. The high incidence of these diseases has prompted the search for novel alternatives for their prevention and treatment. Interestingly, the pathogenesis and development of several NCDs have been linked to an excessive formation and accumulation of advanced glycation end products (AGEs) [5,6]. AGEs are a heterogeneous group of compounds whose synthesis frequently begins with the non-enzymatic glycation of proteins [7]. The abnormal AGEs accumulation in human body tissues produces deleterious effects involving protein dysfunction, which arises from changes in their conformation (in some cases, AGEs may produce cross-links between proteins), function, and half-life. Furthermore, AGEs interaction with receptors (RAGEs) can activate inflammatory pathways and lead to the generation of oxidative stress [8].

Consequently, the search for natural compounds that interfere with the AGEs formation and accumulation or that function as AGEs crosslink-breakers or RAGEs antagonists (all of which are referred to as antiAGEs compounds in this review) may lead to the discovery and development of novel therapies for diseases in which AGEs accumulate excessively [9,10].

Most known natural antiAGEs compounds were originally found in plants and encompass various compounds such as polyphenols, polysaccharides, terpenoids, vitamins, alkaloids, and peptides [11,12,13,14,15,16,17]. Despite the well-known ability of endophytes to produce bioactive compounds and that sometimes they synthesize the same or similar compounds produced by their host plant [18,19], they have been scarcely studied as producers of antiAGEs compounds.

Thus, our aim was to review and highlight the potential of endophytes as natural sources of antiAGEs compounds. Firstly, we briefly discuss the importance of searching for this kind of compounds due to the suggested connection between excessive AGEs accumulation and development of a large number of chronic diseases. Secondly, we summarize some of the reported antiAGEs plant-derived compounds that have been also found as metabolites of endophytes. Finally, we point out the advantages and challenges of using endophytes instead of plants to discover and produce antiAGEs compounds.

## 2. Endophytes, an Exceptional Source of Bioactive Compounds

Endophytes comprise mainly fungi and bacteria, but it also includes archaea and protists that live in the internal tissues and organs of plants (leaves, stems, flowers, fruits, seeds, or roots). Some endophytes do not cause apparent signs of disease in their host plants [20]; other may even be beneficial to their host [7], while some could become opportunistic pathogens under particular circumstances [1]. The above depends on the plant and microbial genotype, quorum sensing, co-colonizing microbiota, and environmental conditions [1,21,22]. Endophyte colonization may occur by horizontal transference through different ways, such as soil-to-root, by phyllosphere (aerial spores) or through vectors (pollinators, arthropods, or sap-feeders), and by vertical or mixed transfer via seeds [23]. Endophyte colonization could involve passive or active mechanisms. In the first one, endophytes get access into a plant tissue through cracks, wounds, or hydathodes. On the other hand, active mechanisms involve the secretion of cell-wall-degrading and other enzymes [1,24]. Once inside the plant, the competent endophytes may spread systematically to reach other different plant tissues, mainly via the xylem vascular system [23].

The diversity and composition of endophytic communities in plants depend on biotic factors such as genotype, developmental stage, and physiology of the host plant. Also, microbial strain type, the endophyte chemotaxis to plant–exudates production, and presence of other microorganisms are involved. In addition, abiotic factors such as soil characteristics (pH, moisture, nutrients, presence of pollutants) and environmental conditions (temperature and radiation) could modify the establishment of endophytic communities [1,25,26].

The endophyte–host plant relationship is diverse, complex, and, in many cases, not totally understood. Endophytes could be mutualistic, commensal, and even opportunistic pathogens [1]. In mutualistic endophyte–host plant relationship, the plant offers shelter and nutrients for microorganism survival. In exchange, endophytes can promote plant growth, induce a plant defense response, improve the nutrient’s availability, increase resistance to biotic (salinity, drought, heat, and cold) and abiotic stress (caused by phytopathogens or herbivores), and consequently, enhance the plant survival [26,27,28].

Some of the interactions mentioned above take place by eliciting host response or by secondary bioactive metabolites produced by the endophytes [1,2,27]. Thus, endophytes synthesize metabolites that may be useful for the host plant, e.g., antifungals, plant-growth promoters, antibiotics, insecticides, antioxidants, and antiparasitic agents. Moreover, several metabolites synthesized by endophytes have shown bioactivities that could be useful in industrial, agricultural, and medical fields, for example, lytic enzymes, antidiabetics, anti-inflammatory, anticancer, immunosuppressives, antivirals, antiacetylcholinesterase, antimalarial, analgesic, etc. [18,27,29]. Additionally, endophytes at times may be able to produce some of the compounds produced by their host plant [19,29]. In summary, the endophytes represent an interesting and environmentally friendly source of potentially valuable bioactive compounds.

## 3. Non-Communicable Diseases, a Global Health Problem

For decades, unhealthy diet, sedentary lifestyle, tobacco usage, and alcohol consumption in people around the world have become the main risk factors for the development of non-communicable diseases (NCDs) [4,30]. In 2019, seven of the ten leading causes of death in the world were NCDs or chronic diseases, and it is estimated that their prevalence will continue rising [31]. Accordingly, the detection, treatment, prevention, and palliative care of all these pathologies constitute a crucial challenge for public world health [4]. Moreover, NCDs such as diabetes, cancer, cardiovascular and respiratory diseases are factor risks that have contributed to the severity and rising number of deaths caused by the emergence of the COVID-19 pandemic [32].

Since the pathogenesis and development of some of the main NCDs have been frequently associated with an excessive formation or accumulation of AGEs or the interaction of AGEs with AGEs receptor (RAGE) [33,34], the development of strategies to limit the accumulation of AGEs may be a new potential alternative for the treatment of some NCDs.

## 4. Advanced Glycation End Products

**Figure 1:**
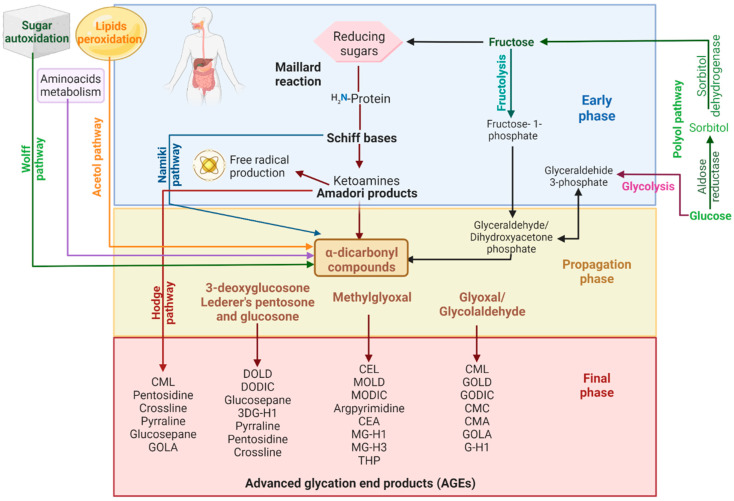
**Endogenous AGEs formation in human body.** AGEs are typically formed in three phases. The early phase involves the Maillard reaction between sugar and proteins and concludes with the formation of Amadori products. Subsequently, the propagation phase generates α-dicarbonyl compounds such as methylglyoxal, glyoxal, and 3-deoxyglucosone. A great variety of AGEs emerge during the final phase. In addition, other pathways that could exacerbate the quantity of AGEs precursors comprise sugar autoxidation, aminoacids metabolism, lipids peroxidation, polyol pathway, fructolysis, and glycolysis. Created with BioRender.com. Adapted from Zeng et al. [6].

AGEs are a heterogeneous group of molecules whose formation usually involves non-enzymatic reactions of reducing sugars with proteins through the Maillard reaction [7]. The endogenous formation of AGEs is shown in Figure 1. Initially, the carbonyl group of a reducing sugar reacts with amino groups of proteins, preferentially those of lysine or arginine, to form Schiff bases [35,36]. Rearrangements of the Schiff bases lead to the formation of more stable compounds known as Amadori products (ketoamines) [35,36]. Subsequently, the Amadori products via oxidation, deprotonation, and fragmentation reactions form dicarbonyl compounds in the propagation phase [35]. Methylglyoxal and other α-dicarbonyl compounds are the primary AGEs precursors. These precursors may also originate from sugar autoxidation, lipid peroxidation, amino acid breakdown, and acetone metabolism. Polyol pathway, glycolysis, and fructolysis are metabolic pathways that may contribute to the triose phosphate pool and consequently to the methylglyoxal formation [37,38,39]. Ultimately, reactions of cyclization, isomerization, retro-aldol cleavage, hydrolytic and oxidative α-cleavage, and β-cleavage may generate a great variety of AGEs in the final phase of AGEs formation [7,38].

Depending on the chemical structure and ability to emit fluorescence, AGEs can be classified as fluorescent and cross-linked, (e.g., pentosidine, crossline, and vesperlysine), fluorescent and non-cross-linked, (e.g., argpyrimidine), non-fluorescent and cross-linked, (e.g., glyoxal-lysine dimer, methylglyoxal-lysine dimer, glyoxal-derived imidazolium cross-link, methylglyoxal-derived imidazolium cross-link, etc.), and non-fluorescent, non-cross-linked adducts, (e.g., carboxymethyl-lysine, carboxyethyl-lysine, pyrraline, and imidazolones) [40]. The cellular formation of AGEs is common under physiological conditions. However, it may undesirably increase under conditions of hyperglycemia, hyperlipidemia, oxidative stress, and inflammation, all of which are common in diabetes, chronic diseases, and ageing [41]. In addition to the endogenous formation of AGEs, exogenous sources such as dietary AGEs (dAGEs) may be consumed from fried or processed foods [42]. Furthermore, AGEs may be inhaled from tobacco smoke, which contributes to the AGEs circulating in the body [43]. Increased rates of AGEs production or accumulation may have pernicious health consequences because AGEs could prompt the formation of covalent cross-links between proteins to form aggregates or may alter the conformation, activity, or function of proteins, as well as their removal by proteolytic means [15,29]. Moreover, AGEs often trigger intracellular signaling processes through their attachment to AGEs receptors (RAGE), so they may cause oxidative stress, inflammatory responses, immune dysfunction, and DNA damage [44,45]. The interactions cited above may explain, at least in part, why AGEs have been linked to a wide range of diseases.

### 4.1. High Levels of AGEs Accumulation Are Linked to Various Diseases

Diabetic patients recurrently show higher blood sugar concentrations than healthy people, which fosters higher levels of AGEs accumulation and AGEs–RAGEs interactions. This has been linked to the pathogenesis of diabetic complications such as retinopathy, cataract, neuropathy, nephropathy, atherosclerosis, and heart diseases [46,47,48]. Additionally, increasing AGEs levels contribute to the progression of neurodegenerative diseases, e.g., Alzheimer’s, Parkinson’s, and Huntington’s diseases, and generates cross-links and consequently prompts the formation of the aggregates with amyloid β and tau proteins, α-synuclein, and huntingtin, respectively, as well as alterations via the AGEs–RAGEs axis [49]. Furthermore, AGEs and p53 proteins have been linked to tumorigenesis in lung, breast, colorectal, pancreatic, and melanoma cancer [50].

**Figure 2:**
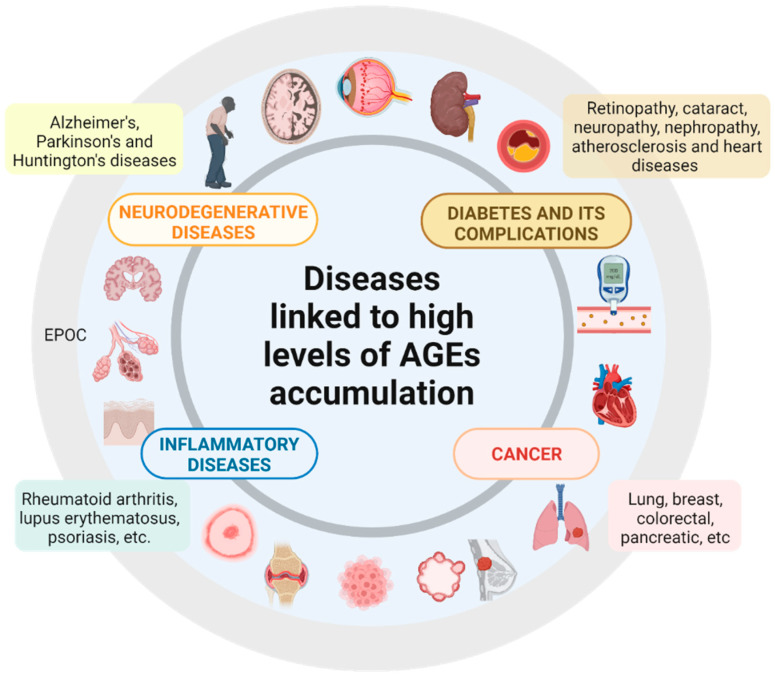
**Diseases linked to high levels of AGEs accumulation.** The excessive formation or accumulation of AGEs and their interaction with RAGEs contribute to the pathogenesis and development of diabetic complications, different kinds of cancer, and neurodegenerative and inflammatory diseases. Created with BioRender.com.

Other diseases that have been associated with high levels of AGEs or AGEs–RAGEs interactions are cardiovascular diseases [51], sarcopenia [52], osteoporosis [53], inflammatory diseases such as rheumatoid arthritis [54], lupus erythematosus [55], psoriasis [56], chronic lower limb ischemia [57] and chronic obstructive pulmonary disease [58] (Figure 2). Recently, it has been reported that activation of the RAGEs axis could exacerbate clinical complications in COVID-19 patients with diabetes [59].

### 4.2. Reducing AGEs Accumulation as a Potential Treatment Strategy for Some NCDs

The high incidence and prevalence of NCDs emphasize the importance of finding new treatment alternatives. It has been proposed that the inhibition of formation or accumulation of AGEs may help to delay or prevent the progression of some non-communicable diseases [10,45,60]. In order to reduce the exogenous AGEs intake, it is often recommended to consume fresh vegetables, fruits, and whole grains, as well as restrict sugary, processed, or fried foods, and cook meals at low temperatures with high humidity. Similarly, having a healthy diet and lifestyle, including exercise and not smoking, are important for the prevention or management of most, if not all, NCDs [61].

Cells possess their own AGEs detoxification systems, e.g., glyoxalase. However, under pathogenic conditions or with ageing, they often become insufficient to keep optimal physiological conditions. Therefore, compounds that inhibit the formation or prevent an excessive accumulation of AGES may represent a potential strategy to retard the onset of detrimental health effects resulting from undue AGEs accumulation and, by doing so, may delay the development of NCDs [10,60].

**Figure 3:**
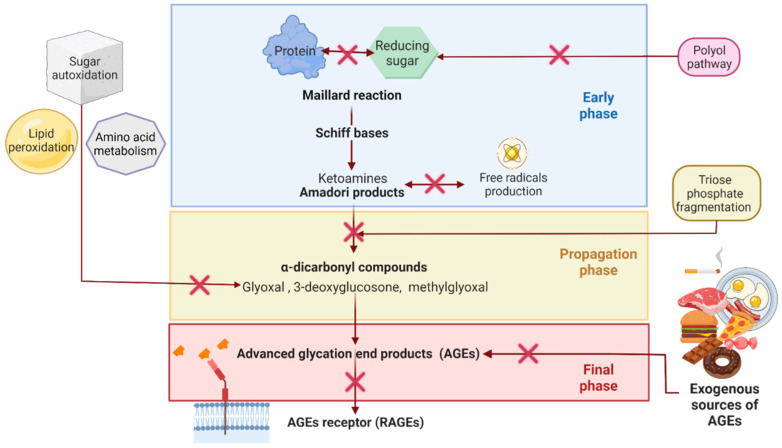
**The mechanisms of action of antiAGEs compounds.** The antiAGEs compounds could restrict in different ways, shown with a red cross, the undue accumulation, and consequent harmful effects of AGEs. These compounds may block sugar attachment to proteins, scavenge free radicals, chelate ions, trap reactive dicarbonyl species, break AGEs cross-links, or block the AGEs–RAGEs interaction. Hyperglycemic control and inhibition of aldose reductase or sorbitol dehydrogenase may decrease the reducing sugars available and, therefore, the formation of AGEs. Created with Biorender.com.

Due to the rather complex AGEs formation process, several mechanisms exist by which a given compound may operate for this purpose. In this review, we refer to “antiAGEs compounds” as those that may reduce the harmful consequences of AGEs accumulation by at least one of the action mechanisms enlisted below (Figure 3):

- Blocking the carbonyl groups of reducing sugars or stabilizing the protein structure to inhibit the Maillard reaction or the formation of Schiff bases and Amadori products;
- Scavenging of free radicals and chelating metal ions. Consequently, fewer reactive carbonyl groups and fewer radical-based reactions occur;
- Blocking or breaking the AGEs cross-links to lessen the protein aggregation;
- Disrupting the AGEs–RAGE interaction, thus preventing inflammatory process and oxidative stress;
- Some indirect mechanisms may be stimulating the glyoxalase system and other dicarbonyl detoxification systems to reduce the available AGEs precursors. Inhibition of polyol pathway enzymes (aldose reductase and sorbitol dehydrogenase) to reduce fructose intake and hypoglycemic activity to reduce sugar availability, etc. [9,60].

### 4.3. Synthetic AntiAGEs Compounds

Synthetic antiAGEs compounds include aminoguanidine, N-phenacylthiazolium bromide (PTB), tenilsetam, pyridoxamine, pentoxifylline, benfotiamine, LR-90, alagebrium chloride (ALT-711), edaravone, TM2002, pioglitazone and metformin [10]. The two last compounds are widely used for diabetes treatment. Edaravone has been used to treat amyotrophic lateral sclerosis [62], whereas pentoxifylline is used to improve blood flow in patients with circulation problems. However, most of the other antiAGEs compounds have failed in human clinical trials due to severe side effects or deficient effectiveness [10]. For that reason, natural antiAGEs compounds are being studied as a potentially safer and environmentally friendly alternative.

Newman and Cragg [63] wrote: “Natural products still hold out the best options for finding novel agents/active templates, which, when worked on in conjunction with synthetic chemists and biologists, offer the potential to discover novel structures that can lead to effective agents in a variety of human diseases”.

Several natural antiAGEs compounds have been found and identified as plant metabolites, including polyphenols, polysaccharides, terpenoids, vitamins, alkaloids, and peptides [11,12,13,14,15,16,17]. In contrast, there are scarce reports about antiAGEs compounds synthesized by endophytes, despite the fact that in some cases, these organisms have the capacity to generate the same or similar bioactive compounds as their host [29,64].

## 5. Plant AntiAGEs Compounds Also Are Found in Endophytes

Endophytes are a rich source of a wide variety of chemical compounds such as alkaloids, phenols, tannins, amino acids, carbohydrates, saponins, terpenes, flavonoids, and sterols [65]. Various metabolites and crude extracts of endophytes have shown antioxidant activity, which is known as a possible mechanism for inhibiting the formation of AGEs [66,67]. Gutiérrez-García et al. [68] explored the antiAGEs compounds produced by endophytes from *Piper auritum*. They found that 2,4-diacetylphloroglucinol (DAPG) and congeners such as 5-hydroxyferulic acid synthesized by endophytic *Pseudomonas* strains inhibit, in vitro, the formation of Amadori products and fluorescent-AGEs.

Natural antiAGEs compounds have been studied and found primarily in plants. However, some of these plant-derived antiAGEs compounds have also been found as metabolites synthesized by endophytes [13,29,69,70]. Table 1 summarizes some plant anti-AGEs compounds, their mechanism of action, the endophytes reported to be capable of producing them, and the analytical techniques used to identify these compounds in the endophytes. Table 1 was originally built from the examination of the four most-cited reviews, and three recent reviews, on inhibition of advanced glycation end products by natural products [11,12,13,14,15,16,17]. This led us to identify ~130 natural compounds with at least one antiAGEs activity. Each compound was separately searched in the Web of Science (Core Collection) using the compound name and the phrase “Advanced glycation end products”. Next, the compounds with at least six outputs (62) were searched by name and the input “endophyte”. We retrieved 382 outputs but only 70 were about the production of the antiAGEs compounds by endophytes, and they included ~30 antiAGEs compounds. Only the papers that included reliable spectrometric data and chemical information about the compounds synthesized by endophytes were further considered. Table 1 summarizes 26 compounds plus some derivatives, 47 papers about the mechanisms of action involved in the antiAGEs activity of the compounds cited, and 37 articles reporting endophytes that synthesize at least one of the mentioned compounds.

As is shown in Table 1, many antiAGEs compounds synthetized by endophytes have been reported. However, we must be very careful because the identity of a compound should be confirmed, if possible, using complementary methods such as NMR, IR, MS, etc. When working with endophytes, is often difficult to obtain enough quantity of a pure compound or the standard to confidently determine its identity and structure. For this reason, the compound’s identity is often assigned based on databases and literature comparisons. Unfortunately, this is often a complex undertaking due to the varied operation conditions of the analytical instruments, (e.g., experimental vs. those used in databases) or spectra similarities existing among compounds of the same type. For example, paclitaxel could give similar UV spectra, retention times in chromatography, and even m/z signals in mass spectrometry to other different taxanes [155]. For small molecule elucidation using high-resolution mass spectrometry, a levels system approach has been proposed to improve the confidence in identification. In this system, level 1 represents the ideal situation where the proposed structure is confirmed via measurement of a reference standard with MS/MS [156]. The above emphasizes the need for further research to determine the ability of endophytes to synthesize some of the antiAGEs plant-derived compounds covered here.

### 5.1. Plant-Derived AntiAGEs Polyphenols Reported in Endophytes

**Figure 4:**
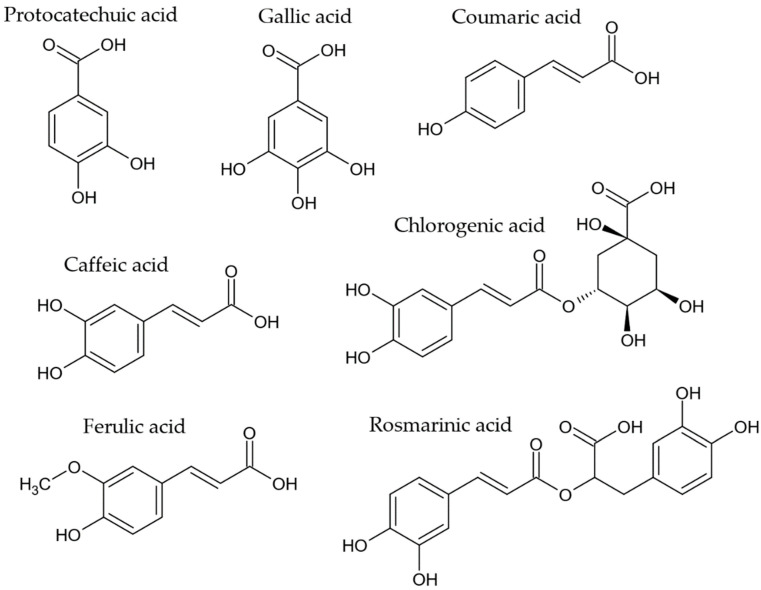
AntiAGEs phenolic acids reported as metabolites in endophytes.

Many of the compounds that curtail the generation of AGEs are classified as polyphenols, characterized by having an aromatic ring with one or more hydroxyl substituents. These natural compounds are often found in plants and include phenolic acids, flavonoids, stilbenes, curcuminoids, and coumarins [13,157,158]. Figure 4 shows some antiAGEs phenolic acids that have been reported to be produced by endophytes such as protocatechuic acid [73,74], gallic acid [78,79,80,81], coumaric acid [80,81], caffeic acid [78,80,81,88,89,90,91], ferulic acid [73,78,81,88], rosmarinic acid [78,81,98], and chlorogenic acid [80,81,89,99].

**Figure 5:**
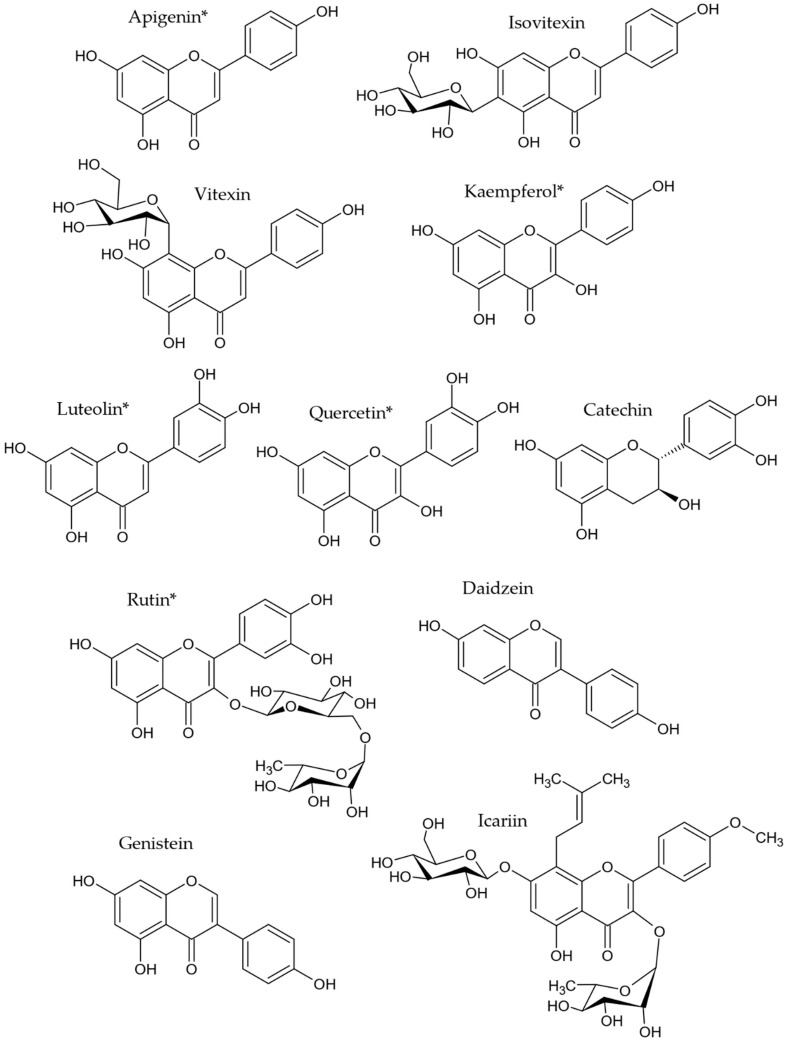
AntiAGEs flavonoids reported in endophytes. * Endophytes are also able to produce derivatives of these compounds.

Flavonoids are polyphenolic compounds well known for their beneficial effects on health, some of them have shown antiAGEs activity [12,157,158]. Flavonoids can be classified into isoflavones, flavones, flavanones, flavonols and anthocyanins. Figure 5 shows some antiAGEs flavonoids reported as metabolites in endophytes consisting of: apigenin and derivatives such as vitexin and isovitexin [78,81,90,103,104,105], kaempferol and derivatives [91,110,111], luteolin [78,81,113], quercetin and derivatives [81,88,90,91,113], catechin [78], daidzein [118], genistein [90,118], icariin [78] and rutin and derivatives [78,81,91].

**Figure 6:**
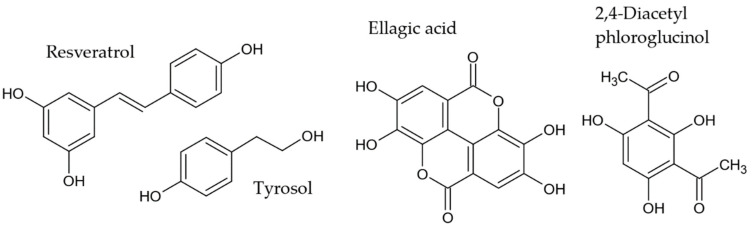
Other antiAGEs phenolic compounds reported in endophytes.

Other antiAGEs phenolic compounds that have been reported in endophytes such as resveratrol, a stilbene [124,125,126,127,128], tyrosol [131,132,133,134], ellagic acid [80,91], and 2,4-diacetyl-phloroglucinol [68] are shown in Figure 6.

### 5.2. AntiAGEs Terpenoids Reported as Metabolites from Endophytes

**Figure 7:**
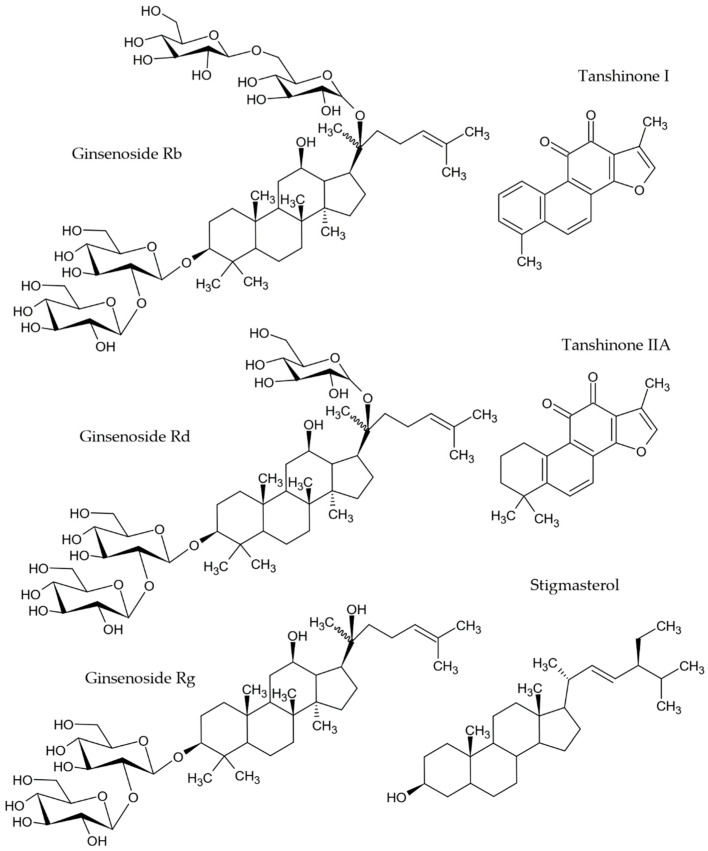
AntiAGEs terpenoids reported in endophytes.

Terpenoids and isoprenoids are among the most abundant and structurally diverse group of plant natural products; however, endophytes are becoming an increasingly recognized source of these compounds [159]. Some antiAGEs terpenoids reported in endophytes (Figure 7) comprise ginsenosides (Rb, Rd, Rg) [137,138,139], tanshinones [142], and stigmasterol [144].

### 5.3. Other AntiAGEs Compounds Reported in Endophytes

**Figure 8:**
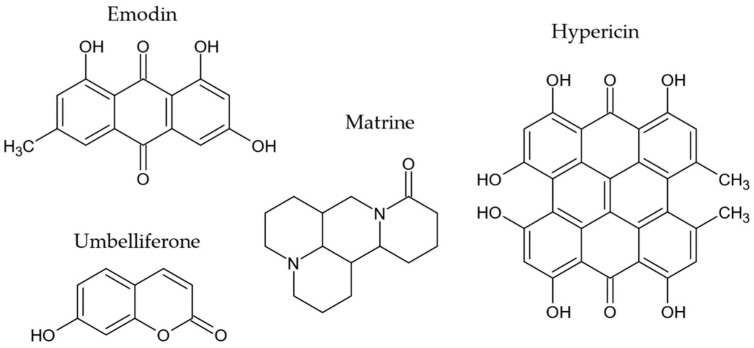
Other antiAGEs compounds reported in endophytes.

In addition, other antiAGEs compounds of different chemical classes have been reported in endophytes (Figure 8), such as the anthraquinone, emodin [146,147,148], the hydroxycoumarin, umbelliferone [80], the naphthodianthrone, hypericin [148] and the alkaloid, matrine [152].

## 6. Endophytes May Encourage the Production of antiAGEs Compounds by Plants

Endophyte inoculation in plants could change through unique interactions, their own secondary metabolite synthesis, or the metabolites produced by the host plant [160]. In some cases, endophytes may elicit the synthesis of certain plant compounds, such as alkaloids [161] and phenolics [162]. Berberine is an isoquinoline alkaloid with antiAGEs activity [163]. Their synthesis in *Coptis teeta* was positively linked with the presence of *Microbacterium* species [164]. *Phialocephala fortinii*, an endophyte from *Rhododendron pseudochrysanthum*, prompted an increase of rutin, hyperoside, quercitrin, and catechin in seedlings [162]. Various other endophytes have elicited the production of tanshinones in *Salvia miltiorrhiza* [165].

Moreover, endophytes may modify the compounds produced by their host, giving rise to compounds with distinctive properties and/or bioactivities [19]. For example, *Phomopsis* sp., an endophyte recovered from *Pinus taeda*, biotransformed limonene to produce α-terpineol, carvone, limoneno-1,2-diol, and other limonene derivatives [166]. *Paraconiothyriu variabile*, an endophyte of *Cephalotaxus harringtonia*, biotransformed the host-glycosylated apigenin and chrysoeriol flavonoids into their respective aglycones [167]. *Epicoccum nigrum*, an endophyte of *Salix* sp., was reported to biotransform the host flavonoids into a new kaempferol O-diglycoside [168]. The above examples highlight potential and interesting uses of endophytes to conceivably produce novel antiAGEs compounds.

## 7. AntiAGEs Compounds Production: Endophytes vs. Plants

Endophytes may have some advantages compared to plants for producing bioactive compounds, as follows:

- Shorter production time. Microorganisms grow much faster than plants. Consequently, metabolite mass manufacturing with endophytes may be achieved in shorter periods compared to plants.
- Environmentally friendly. Culturing microorganisms does not require the use of large land areas. This averts overharvesting and reduces dependence on plant biodiversity.
- Reliable metabolite production throughout the year. Endophyte-based metabolite production does not depend on seasonal growth, in contrast to plants, nor on weather fluctuations or geographical conditions. Secondary metabolites could be produced at any time of the year with endophytes.
- More economical process. Usually, microbial sources of valued products are cheaper because they can be mass-produced; this may have an impact on the market price of the compounds of interest.
- Fewer purification steps. The chemical complexity of microorganisms is relatively lower than that of plants. A less complex chemical profile of endophytes makes the purification process of metabolites easier and potentially less costly [3,103,169].

## 8. Challenges for the Future Use of Endophytes as Sources of AntiAGEs Compounds

Despite the possible advantages of endophytes as sources of bioactive compounds (compared to plants), a number of constraints still exist that require diligent consideration to take advantage of endophytes as sources for the discovery and commercial production of antiAGEs compounds. Some constraints may include, for example, (i) numerous endophytes are non-cultivable or become unstable under lab conditions and (ii) low growth yields and a reduction of secondary metabolite production when axenic monocultures are performed [170,171].

Moreover, most studies about bioactive compounds of endophytes have been done under axenic monoculture conditions. However, it is well known that endophytes, in their natural habitat, interact with other organisms, including their host plant and their metabolites, possibly leading to regulation of their own secondary metabolism and cooperating roles for producing bioactive compounds [19]. For that reason, it may be crucial to study the endophytes in systems nearby to their natural habitat because this could disclose the whole potential of endophytes communities to produce bioactive metabolites.

Fortunately, new techniques and technologies, biotechnological platforms, and omic sciences could help to better analyze and understand endophytes and their usually complex interactions with other organisms. In addition, genome mining, genetic engineering, and process optimization (elicitor addition, solid sorbent use, and co-culture fermentation) will allow us to improve the yield and productivity of antiAGEs compounds synthesis by endophytes [19,170,171].

## 9. Conclusions

Endophytes synthesize a wide variety of metabolites, including some specific of their host plant; as such, they seem to be a promising source of antiAGEs compounds. Additionally, endophytes may be useful to elicit the production of bioactive phytochemicals by plants and induce the production of novel ones. However, some outstanding challenges still limit the discovery and commercial use of these microorganisms as sources of antiAGEs compounds and other bioactive compounds. The use of new technologies in biotechnological platforms and the advancement of omic sciences will help in the understanding of endophytes and their complex interactions with other organisms. This new knowledge will allow endophytes to be harnessed as a safe, sustainable, economical, and profitable option for developing new antiAGEs and other pharmaceutic compounds. The above could be a significant aid for the treatment and control of at least some prevalent non-communicable diseases that threaten global health.

## Figures and Tables

**Table 1:** Anti-AGE compounds reported as metabolites synthesized by endophytes.

AntiAGEs Compound	Concentration/Study Model	Action Mechanism	Endophytic Source/Host Plant	Analytical Method of Identification
Protocatechuic acid	* 2–4% in powder diet of T2D rats [71] * 50–100 mg/kg p.o in T2D rats with high fat diet [72]	Reduces formation of CML, pentosidine and the expression of aldose reductase, sorbitol dehydrogenase, and RAGEs. Improves glyoxalase I expression and insulin sensibility. It has antioxidant, hypoglycemic and anti-inflammatory activity [71,72]	-NID endophytes/*Newbouldia laevis* and *Ocimum gratissimum* [73] -*Aspergillus* sp., FVL2/*Foeniculum vulgare* [74]	-HPLC-PDA ^a^ -^1^H, ^13^C, HSQC, and HMBC NMR ^a^
Gallic acid	* 50–200 µg/mL in BSA-glucose system [75] * 25 mg/kg/day in rats [76] * 100 µM in BSA-glucose, BSA-ribose and BSA-MGO system [77]	Diminishes fluorescent AGEs formation and RAGEs expression. Chelates ion metals entrap carbonyl species and have antioxidant, anti-inflammatory and hypoglycemic activities [75,76,77]	-*Fusarium* sp./*Fritillaria unibracteata* [78] -*Alternaria* spp., *Penicillium* spp., *Neurospora* spp., *Cladosporium* spp., *Phoma* spp. *Fusarium* spp., *Phomopsis* spp. and *Pleosporales* spp./*Acer ginnala* [79] -*Cladosporium velox*/*Tinospora cordifolia* [80] -*Fusarium* spp./*Ferula assa-foetida* [81]	-HPLC-DAD ^b^ -HPLC-dual λ detector * -HPLC-DAD ^b^ -HPLC-PDA *
Coumaric acid	* 0.2 mM in rat hepatocytes [82] * 20 mM in rat tail tendons [83]	Decreases collagen cross-links and cytotoxicity induced by GO and MGO in hepatocytes, has antioxidant and anti-inflammatory activity [82,83]	-*Cladosporium velox*/*Tinospora cordifolia* [80] -*Fusarium* spp./*Ferula assa-foetida* [81]	-HPLC-DAD ^b^ -HPLC-PDA *
Caffeic acid	* 0.5–2 mM in BSA-MGO and histones-MGO system [84] * 2.5–5% in powder diet of T2D rats [85] * 10 µM in human endothelial cells system [86] * 0.1–2.5 mM BSA-MGO, HUVEC system [87]	Reduces the levels of CML, fluorescent AGEs and inflammatory hormones. Inhibits aldose reductase, sorbitol dehydrogenase activity and RAGEs expression, has antioxidant, anti-inflammatory activities [84,85,86] * There are contradictory reports about the beneficial effect of caffeic acid. Wu et al. [87] reported proglycation effect of caffeic acid, which leads to the elevation of oxidative stress and inflammation in monocytes, macrophages and vascular endothelial cells *	-Four *Fusarium* spp./*Fritillaria unibracteata* [78] -*Fusarium chlamydosporum* and *Penicillium canescens*/*Polygonum chinense* L. [88] -*Colletotrichum acutatum* S216/*Camellia* [89] -*Arcopilus cupreus*/*Schleichera oleosa* [90] -*Aspergillus fumigatus*/*Moringa oleífera* [91] -*Cladosporium velox/Tinospora cordifolia* [80] -*Fusarium* spp./*Ferula assa-foetida* [81]	-HPLC-DAD ^b^ -HPLC-ESI-MS/MS ^b^ -UPLC-MS/MS ^b^ -OHR-LC-MS (ESI and APCI) ^a^ -UHPLC-DAD ^b^ -HPLC-DAD ^b^ -HPLC-PDA *
Ferulic acid	* 50–200 µg/mL in BSA-glucose system [75]. * 0.2 mM in rat hepatocytes [82] * Equimolar or a 5-fold molar excess with respect to the lysine content of flour and egg white in cake [92]. * 12.95 mM in BSA-fructose or soy glycinin–fructose system [93] * 5–20 mM in BSA-glucose system; 0.1 and 0.2 mM in HUVEC system [94]	Inhibits production of CML, fluorescent AGEs, dicarbonyl compounds, CEL, and melanoidins. Decreases cytotoxicity induced by GO and MGO in hepatocytes, reduces protein cross-linking and has antioxidant, anti-inflammatory and antihyperglycemic activities [75,82,92,93,94]	-NID endophytes/*Newbouldia laevis* and *Ocimum gratissimum* [73] -Three *Fusarium* spp./*Fritillaria unibracteata* [78] -*Fusarium chlamydosporum* and *Penicillium canescens*/*Polygonum chinense* L. [88] -*Alternaria tenuissima* SBUp1, *Fusarium* sp.*/Ferula assa-foetida* [81]	-HPLC-PDA ^a^ -HPLC-DAD ^b^ -HPLC-ESI-MS/MS ^b^ -HPLC-PDA *
Rosmarinic acid	* 6.25–400 µg/mL in BSA-glucose, BSA-GO and BSA-MGO system [95] * 10 µM in HSA–MGO system [96]	Inhibits formation of fluorescent AGEs, CML, and CEL. Reduces MGO levels, protein aggregation, and fibril formation induced by AGEs in human serum albumin [95,96]	-Two *Fusarium* spp./*Fritillaria unibracteata* [78] -*Alternaria tenuissima* SBUp1, *Fusarium* sp./*Ferula assa-fotida* [81]	-HPLC-DAD ^b^ -HPLC-PDA ^b^
Chlorogenic acid	* 0.5–2 mM in BSA-MGO and histones-MGO system [84] * AGEs IC_50_ = 148.32 ± 3.13 µM in BSA-glucose system; crosslinking IC_50_ = 0.68 ± 0.10 mM in AGEs-BSA-rat tail tendon collagen system; carbonyl trapping IC_50_ = 48.26 ± 16.98 mM [97]	Inhibits production of fluorescent AGEs and alpha glycosidases. Reduces cross-linking of AGEs-BSA to collagen, entraps MGO, has antihyperglycemic and antioxidant activities [84,97]	-*Sordariomycete* sp./*Eucommia ulmoides* [98] -*Cochliobolus lunatus*/*Mirabilis jalapa* L. [99] -*Brevibacillus borstelensis* B14, *Bacillus amyloliquefaciens* B17, *Bacillus badius* B19, *Sphingomonas yabuuchiae* N21, *Enterobacter tabaci* N22, and *Lodderomyces elongisporus* P212 and *Colletotrichum acutatum* S216/*Mentha haplocalyx* (B14, B17, B19), *Ipomoea batatas* and *Camellia* [89] -*Cladosporium velox/Tinospora cordifolia* [80] -*Fusarium* sp./*Ferula assa-foetida* [81]	-HPLC, UPLC-PDA-QTOF-MS ^b^ -LC-ESI-MS/MS -Chromogenic method, TLC ^b^, HPLC-UV ^b^, UPLC-MS/MS ^b^ -HPLC-DAD ^b^ -HPLC-PDA *
Apigenin (A) and derivatives (V = vitexin and I = isovitexin)	* AGEs IC_50_ = 85.2–185.2 µM in BSA-glucose-fructose system; aldose reductase IC_50 A_ = 2.47–6.67 µM in RLAR system [100] * Aldose reductase IC_50 V_ = 1.47 ± 0.08 µM, IC_50 I_ = 0.49 ± 0.08 µM, IC_50 A_ = 0.97 ± 0.26 µM in RLAR system and IC_50 V_ = 12.07 ± 0.03 µM, IC_50 I_ = 0.13 ± 0.03 µM, IC_50 A_ = 11.65 ± 0.07 µM in HRAR system; AGES IC_50 V_ = 243.54 ± 8.86 µM, IC_50 I_ = 175.66 ± 3.73 µM, IC_50 A_ = 204.14 ± 9.31 µM in BSA-fructose-glucose system [101] * 10–25 µM IN AGEs-HUVECs system [102]	Inhibits aldose reductase and acetylcholinesterase activities, as well as the formation of fluorescent AGEs. Entraps MGO and reduces inflammatory cytokines and adhesion molecules, has antioxidant and anti-inflammatory activities [100,101,102]	-*Dichotomopilus funicola*/*Cajanus cajan* L. (pigeon pea) [103] -Two *Fusarium* spp./*Fritillaria unibracteata* [78] -*Fusarium solani*/*Cajanus cajan* [104] -*Chaetomium globosum/Cajanus cajan* [105] -*Arcopilus cupreus*/*Schleichera oleosa* [90] -*Alternaria tenuissima* SBUp1, *Fusarium* sp./*Ferula assa-foetida* [81]	-HPLC-ESI-MS ^b^ -HPLC-DAD ^b^ -HPLC-UV-Vis *, LC-MS-ESI ^b^, ^1^H, ^13^C NMR ^a^ -HPLC-MS/MS ^a^ -OHR-LC-MS (ESI and APCI) ^a^ -HPLC-PDA *
Kaempferol and derivatives	* 2–4 mg/kg b.w/day in rats; 1–5 µM in YPEN cells [106]. * Scavenging activity IC_50_ = 39.5–55.5 µM [107] * IC_50_ = 10 µM in RLAR system [108] * 20 mg/kg/day in diabetic rats [109]	Inhibits aldose reductase and entraps dicarbonyl compounds. Reduces AGEs levels and hyperglycemia, suppressing AGEs–RAGEs axis activation. It has antioxidant and anti-inflammatory activities [106,107,108,109]	-*Mucor fragilis*/*Sinopodophyllum hexandrum* [110] -*Penicillium setosum*/*Withania somnifera* [111] -*Aspergillus fumigatus*/*Moringa oleífera* [91]	-TLC ^b^, HPLC-UV ^b^, ^1^H, ^13^C NMR ^a^ -HPLC-UV-Vis-Q-ToF-ESI-MS ^a^ -UHPLC-DAD ^b^
Luteolin and derivatives	* AGEs IC_50_ = 16.5–88.9 µM in BSA-glucose-fructose system; aldose reductase IC_50 A_ = 0.087–0.94 µM in RLAR system [100]. * 100 µg/mL in BSA-glucose-fructose system [112]	Inhibits aldose reductase, and production of pentosidine and other fluorescent AGEs. Reduces protein cross-linking [100,112]	-*Nigrospora oryzae*/*Loranthus micranthus* [113] -*Fusarium* sp./*Fritillaria unibracteata* [78] -*Alternaria tenuissima* SBUp1, *Fusarium* sp./*Ferula assa-foetida* [81]	-HPLC-DAD-ESI-MS ^a^, ^1^H, ^13^C, HSQC, and HMBC NMR -HPLC-DAD ^b^ -HPLC-PDA *
Quercetin and derivatives	* 50–200 µg/mL in BSA-glucose system [75] * 100 µM in BSA-glucose, BSA-ribose and BSA-MGO system [77] * AGEs gral IC_50_ = 65 µM, Pentosidine IC_50_ = 18 µM and 75–300 mM in collagen-glucose system [114] * 0.5–2.5 mM in BSA-MGO and BSA-GO system [115]	Inhibits aldose reductase, and the formation of alpha dicarbonyl compounds, CML, and fluorescent AGEs. Entraps MGO and GO, and reduces cross-linking of proteins and glucose autooxidation, chelates metal ions, has antioxidant activity [75,77,108,114,115]	-*Nigrospora oryzae*/*Loranthus micranthus* [113] -*Fusarium chlamydosporum* and *Penicillium canescens*/*Polygonum chinense* L. [88] -*Penicillium setosum*/*Withania somnifera* [111] -*Arcopilus cupreus*/*Schleichera oleosa* [90] -*Aspergillus fumigatus*/*Moringa oleífera* [91] -*Alternaria tenuissima* SBUp1, *Fusarium* sp./*Ferula assa-foetida* [81]	-HPLC-DAD-ESI-MS ^a^, ^1^H, ^13^C, HSQC, and HMBC NMR -HPLC-ESI-MS/MS ^b^ -HPLC-UV-Vis-Q-ToF-ESI-MS ^a^ -OHR-LC-MS (ESI and APCI) ^a^ -UHPLC-DAD ^b^ -HPLC-PDA *
Catechin	* 50–200 µg/mL in BSA-glucose system [75] * 100 µM in BSA-glucose, BSA-ribose and BSA-MGO system [77] * AGEs IC_50_ = 0.049 ± 0.019 mg/mL in BSA-glucose system, radical scavenging IC_50_ = 7.927 ± 0.007 and 5 mM for MGO scavenging	Inhibits the formation of fluorescent AGEs. Chelates metal ions, entraps dicarbonyl compounds, and has antioxidant activity [75,77,116]	-*Fusarium* spp./*Fritillaria unibracteata* [78]	-HPLC-DAD ^b^
Daidzein	* 1 mM in MGO system [117]	Entraps MGO [117]	-*Rahnella aquatilis*/*Emilia sonchifolia* [118]	-ESI-MS ^a^, ^1^H, ^13^C NMR ^a^
Genistein	* 100 µM in BSA-glucose, BSA-ribose and BSA-MGO system [77] * 1 mM in MGO system [117]	Chelates metal ions, entraps MGO, has antioxidant activity [77,117]	-*Rahnella aquatilis*/*Emilia sonchifolia* [118] -*Arcopilus cupreus*/*Schleichera oleosa* [90]	-ESI-MS ^a^, ^1^H, ^13^C NMR ^a^ -OHR-LC-MS (ESI and APCI) ^a^
Icariin	* 20 mg/kg/day in diabetic rats [119] * 10 and 20 mg/kg b.w. in diabetic rats [120]	Reduces blood glucose levels in diabetic rats, has antioxidant, anti-inflammatory and antihyperglycemic activities [119,120]	-*Fusarium* spp./*Fritillaria unibracteata* [78]	-HPLC-DAD ^b^
Rutin and derivatives	* 50–100 mg/kg body weight in diabetic rats (review) [121]	Inhibits alpha-glucosidases, alpha-amylases, aldose reductase, intestinal carbohydrate absorption, and AGEs formation. Increases glucose uptake and insulin secretion. Reduces activity of enzymes involved in gluconeogenesis and has antioxidant and anti-inflammatory activities [121]	-*Fusarium* spp./*Fritillaria unibracteata* [78] -*Aspergillus fumigatus*/*Moringa oleífera* [91] -*Alternaria tenuissima* SBUp1, *Fusarium* sp./*Ferula assa-foetida* [81]	-HPLC-DAD ^b^ -UHPLC-DAD ^b^ -HPLC-PDA *
Resveratrol	* 5 mg/kg b.w. in diabetic rats [122] * 50–300 μg/mL in BSA-fructose, BSA-MGO and arginine-MGO system; α-amylase IC_50_ = 3.62 μg/mL and α-glucosidase IC_50_ = 17.54 μg/mL [123]	Inhibits aldose reductase, alpha-glucosidase, alpha-amylase, and sorbitol dehydrogenase. Chelates metal ions and entraps dicarbonyls. Improves insulin sensitivity, glyoxalase-I activity, and adiponectin levels. Reduces AGEs levels in diabetic rats, has antioxidant and anti-inflammatory activities [122,123]	-*Alternaria* spp., *Botryosphaeria* sp., *Penicillium* spp., *Cephalosporium* spp., *Aspergillus* sp., *Geotrichum* sp., and *Mucor* sp./*Vitis vinifera* L. cv. Merlot, *Vitis quinquangularis* and *Polygonum cuspidatum* [124] -*Arcopilus aureus*, *Penicillium* spp., *Lasiodiplodia* spp., *Nigrospora* sp., *Botryosphaeria* spp., *Fusarium* spp., *Xilaria* sp., *Aspergillus* spp. and *Alternaria* sp./*Vitis vinifera* [125,126,127] -*Aspergillus niger*/*Vitis vinifera* Cabernet Sauvignon [128]	-HPLC-dual λ * -Biochemical assays, TLC ^b^, HPLC * -Chromogenic method, TLC ^b^, UV spectra ^b^, LC *
Tyrosol	* 5–20 mg/kg b.w. in normal and diabetic rats [129] * α-glucosidase IC_50_ = 70.8 µg total phenolic/mL [130]	Inhibits alpha-glucosidase, relieves hyperglycemia, and has antioxidant activity [129,130]	-*Rhytismataceae* sp./*Picea mariana* [131] -*Papulaspora immersa*/*Smallanthus sonchifolius* [132] -*Phialocephala fortinii*/*Rhodiola angusta* and *R. crenulata* [133] -*Pestalotiopsis microspore*/*Manilkara zapota* [134]	-HPLC ^a^, ^1^H, ^13^C NMR ^a^ -Optical rotation, IR, ID, and 2D NMR and MS data ^a^ -HPLC-UV ^b^, UPLC/Q-ToF-MS, and ^1^H-NMR ^b^ ^−1^H, ^13^C NMR ^a^, and FABMS ^a^
Ellagic acid	* Aldose reductase IC_50_ = 0.27 µM in HRAR and IC_50_ = 0.047 µM in RLAR system [108]	Inhibits aldose reductase and sorbitol dehydrogenase activities. Reduces production of CEL, CML, and fluorescent AGEs. Entraps dicarbonyl compounds. Enhances insulin signaling, adiponectin receptors, glucose transporters, and inflammatory mediators. Decreases blood glucose levels and has anti-inflammatory activity [108,135]	-*Cladosporium velox*/*Tinospora cordifolia* [80] -*Aspergillus fumigatus*/*Moringa oleífera* [91]	-HPLC-DAD ^b^ -UHPLC-DAD ^b^
Ginsenosides (Rb, Rd, Rg)	* AGEs IC_50_ = 15–220 µM in BSA-fructose-glucose system [136]	Inhibits production of fructosamine, fluorescent AGEs, and CML. Reduces levels of amyloid cross-B structure, has hypoglycemic activity [136]	-*Fusarium* sp. and *Aspergillus* sp./*Panax notoginseng* [137] -*Fusarium* spp., *Aspergillus* spp., *Verticillium* spp., *Penicillium* spp., *Nectria* spp., and *Plectosphaerella* sp./*Panax ginseng* [138] -*Penicillium* sp., *Dictyochaeta* sp. and *Camarosporium* sp./*Aralia elata* [139]	-HPLC-UV, HPLC-ESI-MS ^b^ -HPLC-PAD ^b^ -HPLC ^b^
Tanshinones	* 5 and 20 mg/kg/day in transgenic mice [140] *10 mg/kg/day in diabetic rats [141]	Reduces plasma glucose, AGEs levels, and RAGE expression. Suppress the activation of NF-κB signaling pathway mediated by RAGE, has anti-inflammatory activity [140,141]	-*Trichoderma atroviride*/*Salvia miltiorrhiza* [142]	-HPLC-HRMS/MS ^b^
Stigmasterol	* 0.1 mg/mL in BSA-glucose system [143]	Inhibits formation of fluorescent AGEs and protein glycoxidation. Entraps carbonyl intermediates, blocks lysyl residues of BSA, and consequently reduces its binding with glucose. It has antioxidant activity [143]	-*Cunninghamella* sp./*Salicornia bigelovii* Torr [144]	-ESI-MS, ^1^H-NMR^a^
Emodin	* AGEs IC_50_ = 118 µM in BSA-fructose-glucose system, aldose reductase IC_50_ = 15.9 µM in RLAR system [145]	Inhibits aldose reductase activity and formation of fluorescent AGEs and CML. Entraps MGO, has antioxidant activity [145]	-*Talaromyces* spp. *Apergillus* spp. and *Fusarium* spp./*Artemisia annua* L. [146] -*Coniochaeta velutina*/*Tsuga heterophylla* [147] -*Thielavia subthermophila*/*Hypericum perforatum* [148]	-Metabolomic analysis by LC-HRMS/MS ^ab^ -LC-MS-IT-TOF and NMR data ^a^ -HPLC-HRMS *
Umbelliferone	* AGEs IC_50_ = 2.95 ± 0.02 µM in BSA-fructose-glucose system [149] * 15–240 µg/mL in psoas muscle system, α-amylase IC_50_ = 8.06 µg/mL	Inhibits production of alpha-glycosidase, alpha-amylase, aldose reductase, fluorescent AGEs, and alpha-dicarbonyl compounds. Improves insulin secretion and glucose uptake, has antioxidant and hypoglycemic activities [149,150]	-*Cladosporium velox/Tinospora cordifolia* [80]	-HPLC-DAD ^b^
Matrine	* 50–100 mg/kg in transgenic mice, 10–50 µM	Inhibits RAGEs activation, has anti-inflammatory activity [151]	-*Aspergillus terreus*/*Sophora flavescens* [152]	-HPLC-PAD ^b^
Hypericin	* 1–10 µM in BSA-MGO system, 0.01–0.5 µM in HUVEC-MGO system [153] * α-glucosidase IC_50_ = 4.66 ± 0.27 mg/L [154]	Inhibits production of α-glucosidase and fluorescent AGEs. Protects against MGO-induced apoptosis and oxidative damage [153,154]	-*Thielavia subthermophila*/*Hypericum perforatum* [148]	-HPLC-HRMS *, detection of *hyp-1* gene

MGO: methylglyoxal; GO: glyoxal; NID: not identified; CML: carboxymethyl lysine; CEL: carboxyethyl lysine; ROS: reactive oxygen species; BSA: bovine serum albumin; HSA: human serum albumin; RLAR: rat lens aldose reductase; HRAR: human recombinant aldose reductase; b.w.: body weight; HUVEC: human umbilical vein endothelial cells; HPLC-PDA: high-performance liquid chromatography coupled to a photodiode array detector; DAD: diode array detector; ESI: electrospray ionization; MS/MS: mass spectrometry in tandem; HRMS: high-resolution mass spectrometry; dual λ: dual wavelength absorbance detector; UPLC: ultra-performance liquid chromatography; ^1^H and ^13^C NMR: proton and carbon nuclear magnetic resonance; HSQC: heteronuclear single quantum coherence NMR; HMBC: heteronuclear multiple bond correlation NMR; OHR-LC-MS: orbitrap high-resolution liquid chromatography coupled to mass spectrometry; APCI: atmospheric pressure chemical ionization; TLC: thin layer chromatography; UV: ultraviolet; IR: infrared spectroscopy; QToF-MS: quadrupole time of flight mass spectrometry; FABMS: fast atom bombardment mass spectrometry; IT: ion trap. ^a^ Comparing with database or literature. ^b^ Comparing with standard data processed under the same conditions. * Quantitative method.

## Abbreviations

CML, Nε-carboxymethyl-lysine; GOLA, Nε-[2-[(5-amino-5-carboxypentyl) amino]-2-oxoethyl]-lysine; DOLD, 3-deoxyglucosonederived lysine dimer; DODIC, 3-deoxyglucosone-derived imidazolium cross-link; 3DG-H1, 3-deoxyglucosone-derived hydroimidazolone 1; CEL, Nε-carboxyethyl-lysine; MOLD, methylglyoxal-derived lysine dimer; MODIC, methylglyoxal-derived imidazolium cross-link; CEA, Nε-carboxyethyl-arginine; MG-H1, methylglyoxal-derived hydroimidazolone 1; MG-H3, methylglyoxal-derived hydroimidazolone 3; THP, tetrahydropyrimidine; GOLD, glyoxal-derived lysine dimer; GODIC, glyoxal-derived imidazolium cross-link; CMC, carboxymethyl cysteine; CMA, Nε-carboxymethyl-arginine; G-H1, glyoxal-derived hydroimidazolone 1.
